# Immune-mediated inflammatory diseases and risk of venous thromboembolism: A Mendelian randomization study

**DOI:** 10.3389/fimmu.2022.1042751

**Published:** 2022-12-13

**Authors:** Xiaoshuo Lv, Xixi Gao, Jingwen Liu, Yisen Deng, Qiangqiang Nie, Xueqiang Fan, Zhidong Ye, Peng Liu, Jianyan Wen

**Affiliations:** ^1^ Department of Cardiovascular Surgery, China-Japan Friendship Hospital, Beijing, China; ^2^ Chinese Academy of Medical Sciences & Peking Union Medical College, Beijing, China; ^3^ Peking University China-Japan Friendship School of Clinical Medicine, Beijing, China

**Keywords:** venous thromboembolism, immune-mediated inflammatory diseases, Mendelian randomization, inflammatory bowel disease, ulcerative colitis

## Abstract

**Introduction:**

Immune-mediated inflammatory diseases (IMIDs) have been associated with an increased risk of venous thromboembolism (VTE) in multiple observational studies. However, a direct causally relation between IMIDs and VTE remains unclear to date. Here, we used Mendelian randomization (MR) analysis to investigate causal associations between IMIDs and VTE.

**Methods:**

We collected genetic data from published genome-wide association studies (GWAS) for six common IMIDs, specifically inflammatory bowel disease (IBD), Crohn’s disease (CD), ulcerative colitis (UC), rheumatoid arthritis (RA), psoriasis (PSO), and systemic lupus erythematosus (SLE); and summary-level data for VTE, pulmonary embolism (PE), and deep vein thrombosis (DVT) from the FinnGen database. Two-sample MR analysis using inverse variance weighting (IVW) was performed to identify causal associations between IMIDs and VTE/DVT/PE, and sensitivity analyses were implemented for robustness.

**Results:**

IVW analysis showed a causal relationship between genetically predicted UC (one type of IBD) and the risk of VTE (OR = 1.043, 95% CI: 1.013-1.073, p = 0.004) and DVT (OR = 1.088, 95% CI: 1.043-1.136, p < 0.001), but we found no evidence of causality between UC and PE (OR = 1.029, 95% CI: 0.986-1.074, p = 0.19). In addition, no associations were observed between total IBD, CD, RA, SLE, or PSO and VTE/DVT/PE. Sensitivity analysis found no evidence for horizontal pleiotropy.

**Conclusion:**

This MR study provides new genetic evidence for the causal relationship between IMIDs and the risk of VTE. Our findings highlight the importance of active intervention and monitoring to mitigate VTE risk in patients with IBD, in particular those presenting with UC.

## 1 Introduction

Venous thromboembolism (VTE), including deep vein thrombosis (DVT) and pulmonary embolism (PE), affects ~10 million people worldwide every year, representing the third most common cardiovascular disease globally (1, 2). The 30-day case fatality rate after VTE diagnosis is 10.6%, with about 30% to 50% of survivors developing long-term complications that increase the burden of this disease (3–5).

The formation of venous thrombosis involves a complex pathophysiological process that is triggered by the interaction of multiple risk factors. An increasing number of studies suggest that inflammation is closely associated with VTE (6). Specifically, the activation of the immune system induces a process known as immunothrombosis (7), in which activated immune cells (such as neutrophils and monocytes) interact with platelets and the coagulation cascade, which ultimately leads to thrombosis (8).

Immune-mediated inflammatory diseases (IMIDs) comprise a wide range of conditions, such as inflammatory bowel disease (IBD – e.g., Crohn’s disease – CD and ulcerative colitis – UC), rheumatoid arthritis (RA), skin inflammation (e.g., Psoriasis – PSO), and connective tissue disease (e.g., Systemic Lupus erythematosus – SLE) (9–11). Recent cross-sectional studies have found a higher incidence of VTE in the overall IMID population than in the general population (12). In addition, many observational studies have reported a higher incidence of VTE in patients with IBD (UC and CD) (13, 14), RA (15, 16), PSO (17), and SLE (18), than in those without these diseases.

Despite proposing the existence of a relationship between IMIDs and VTE, most of these studies were limited by observational designs and small sample sizes that hamper effective causal inferences and can be easily affected by several confounding factors. In contrast, Mendelian randomization (MR) analysis is an emerging method in epidemiological research that uses genetic variants as instrumental variables (IVs) to help assess the causal effects of exposure factors on outcomes while minimizing the impact of confounding factors and reverse causation (19).

The availability of large-scale genome-wide association studies (GWAS) on IMIDs and cardiovascular disease made it possible to implement MR analysis to investigate the relationship between various diseases. For example, Gao et al. (20) established a causal relationship between SLE and cardiovascular disease, while Li et al. suggested that varicose veins may have a causal role in DVT (21). However, to date, no MR studies focused on the effect caused by IMIDs on VTE risk. To explore this possibility, we implemented a two-sample MR analysis using newly published GWAS data on various IMIDs (including IBD, UC, CD, RA, PSO, and SLE) and VTE (including DVT, and PE).

## 2 Methods

### 2.1 Two-sample MR

**Figure 1:**
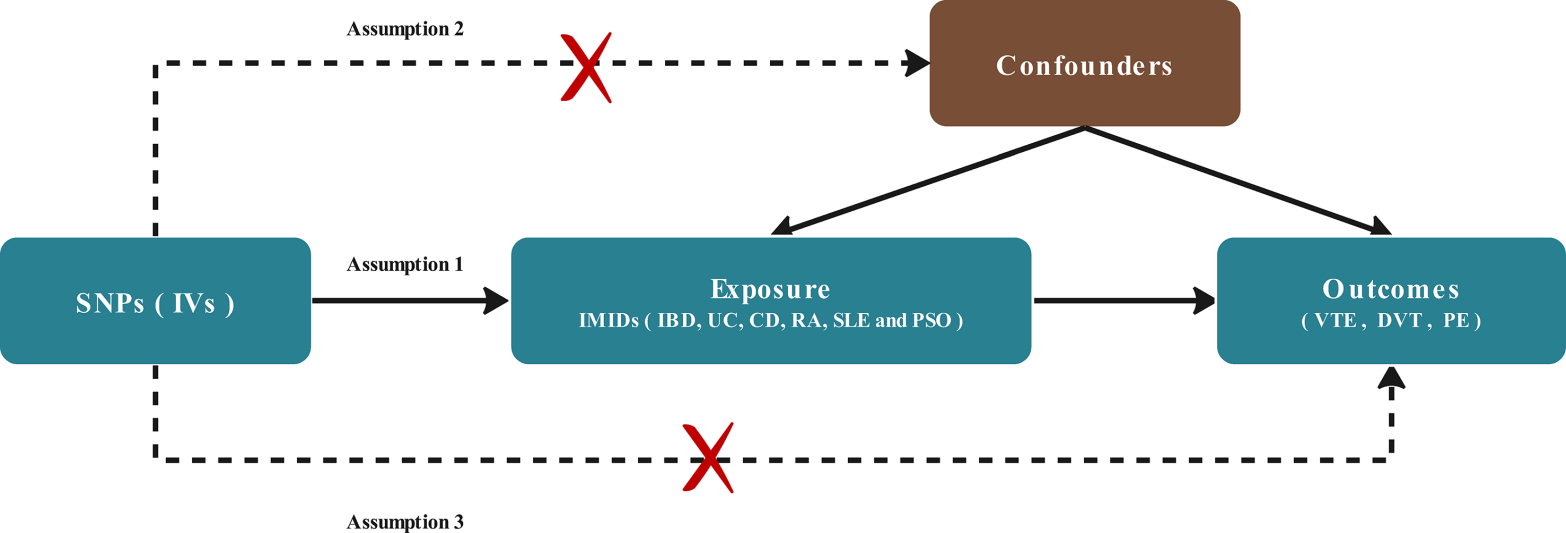
Diagram of the two-sample Mendelian randomization study for the association between IMIDs and risk of VTE/DVT/PE. IVs, instrumental variables; IMIDS, Immune-mediated inflammatory diseases IBD, inflammatory bowel disease; CD, Crohn’s disease; UC, ulcerative colitis; RA, rheumatoid arthritis; SLE, Systemic Lupus erythematosus; PSO, Psoriasis; VTE, Venous thromboembolism; DVT, deep vein thrombosis; PE, pulmonary embolism.

A two-sample MR study was conducted to assess the existence of a causal relationship between genetic susceptibility to VTE (including subtypes of DVT and PE) and IMIDs (including IBD, UC, CD, RA, SLE, PSO). Multiple single-nucleotide polymorphisms (SNP) representing global human genetic variation were selected as instrumental variables (IVs). Three key hypotheses of classical MR analysis were adopted, as follows (**Figure 1**): 1. IVs are directly related to exposure; 2. IVs are independent of any confounding variables; 3. IVs only affect the results *via* exposure (22).

### 2.2 Data sources and study design

Summary-level statistical data for VTE, DVT, and PE were obtained from the latest R7 release of the FinnGen GWAS results (https://r7.finngen.fi/) (23). The corresponding phenotypic codes obtained were “I9_VTE” (14,454 cases and 294,700 controls), “I9_PHLETHROMBDVTLOW” (7,008 cases and 267,090 controls), and “I9_PULMEMB” (6,753 cases and 301,704 controls), respectively. The diagnosis of these cases was determined by the ICD codes.

We retrieved the summary IMIDs GWAS dataset from the IEU OpenGWAS Database Project (https://gwas.mrcieu.ac.uk/). SNPs associated with IBD were obtained from a GWAS study involving 25,042 patients and 34,915 controls, which were further subdivided into secondary outcomes of either UC (12,366 patients and 33,609 controls) or CD (12,194 patients and 28,072 controls) (24). The summary statistics for RA (5,201 patients and 462,933 controls) and PSO (5,314 patients and 462,933 controls) of European descent were obtained from the UK Biobank (http://www.nealelab.is/uk-biobank/). Finally, summary statistics for SLE were derived from a GWAS including 5,201 cases and 9,066 controls (25). The details of the data source and definition are listed in **Table 1**.

**Table 1:** Data sources.

Phenotypes	Data source	phenotypic code	Cases/controls	Ancestry
Exposures				
IBD	de Lange. et al (24)	ebi-a-GCST004131	25042/34,915	European
UC	de Lange. et al (24)	ebi-a-GCST004133	12,366/33,609	European
CD	de Lange. et al (24)	ebi-a-GCST004132	12,194/28,072	European
RA	UK Biobank	ukb-b-9125	5201/457732	European
SLE	Bentham, J. et al (25)	ebi-a-GCST003156	5,201/9,066	European
PSO	UK Biobank	ukb-b-10537	3,871/333,288	European
Outcome				
VTE	FinnGen	I9_VTE	14,454/294,700	European
DVT	FinnGen	I9_PHLETHROMBDVTLOW	7,008/267,090	European
PE	FinnGen	I9_PULMEMB	6,753/301,704	European

IBD, inflammatory bowel disease; CD, Crohn’s disease; UC, ulcerative colitis; RA, rheumatoid arthritis; SLE, Systemic Lupus erythematosus; PSO, Psoriasis; VTE, Venous thromboembolism; DVT, deep vein thrombosis; PE, pulmonary embolism.

### 2.3 Instrumental variable selection

SNPs associated with each IMID at the genome-wide significance threshold *p* < 5.0 × 10^-8^ were selected as potential IVs. To ensure independence between the genetic variants used as IVs, we set the linkage disequilibrium (LD) threshold for grouping to r^2^ < 0.001 and a window size of 1000 kb. The SNP showing the lowest *p*-value at each locus was retained for analyses. The software PhenoScanner (26) was used to examine the associated phenotypes of each genetic variant. SNPs corresponding to the phenotypes associated with VTE were removed to prevent potential pleiotropic effects. In addition, we used the MR residual and outliers (MR-PRESSO) test to detect potential horizontal pleiotropy, and effectively controlled for pleiotropic effects by removing outliers.

Variance (R^2^) and F-statistics were used to estimate the strength of the selected IVs (27). R^2^ was calculated as follows: 2×(1-MAF)×MAF×β^2^ (EAF, effect allele frequency; β, effect size on the exposure). The F-statistic was calculated as follows: F=R^2^(N-K-1)/[K(1-R^2^)], where R^2^ refers to the portion of exposure variance explained by the IVs, N represents the effective sample size, and K represents the number of variants included in the IV model. An F-statistic >10 indicates a strong correlation between the IVs and exposure (28).

### 2.4 Statistical analyses

The “TwoSampleMR” and “MR-PRESSO” packages of the R software (version 4.13) were used to perform MR analysis. We used multiple MR methods to infer causal relationships between a total of six IMIDs and three VTE phenotypes, including inverse variance weighting (IVW) (29), median weighting (30), MR-Egger (31), and MR-pleiotropy residual sum and outlier (MR-PRESSO) (32).

Each method makes different assumptions on the validity of IVs, but the IVW method is generally regarded as the most reliable, so in this study, IVW was used as the primary method to identify the causal relationship between exposure and outcome, while the other methods were used as complementary or to provide other information. IVW is essentially a meta-analysis method combining cumulative causal estimates of Wald ratios for each IV, while MR-PRESSO can automatically detect and remove outliers in IVW linear regression to provide corrected MR estimates (32).

### 2.5 Sensitivity analyses

The Cochrane’s Q test was used to assess the heterogeneity of selected SNPs (P< 0.05), in which case the random effects IVW test was used to provide more conservative and robust estimates. Funnel plots were generated using the mr_funnel_plot function for visualizing the heterogeneity of IVs. In addition, directional pleiotropy was assessed and corrected based on the intercept obtained from the MR-Egger regression model analysis. Finally, we performed a leave-one-out sensitivity analysis to test whether the stability of the results was affected by a single SNP and generated a forest plot to illustrate the results.

## 3 Results

### 3.1 Selection of instrumental variables

SNPs associated with IMIDs were selected as IVs based on established quality control criteria (95 SNPs for IBD, 51 SNPs for UC, 76 SNPs for CD, 5 SNPs for RA, 40 SNPs for SLE, 20 SNPs for PSO). The F-statistics of the vast majority of these SNPs were above the threshold of 10, which indicated that they strongly represent IMIDs in the MR analysis. The detailed characteristics of these IVs are displayed in **Supplementary Table S1**.

### 3.2 Causal estimates of genetic susceptibility to IMIDs and VTE risk

**Figure 2:**
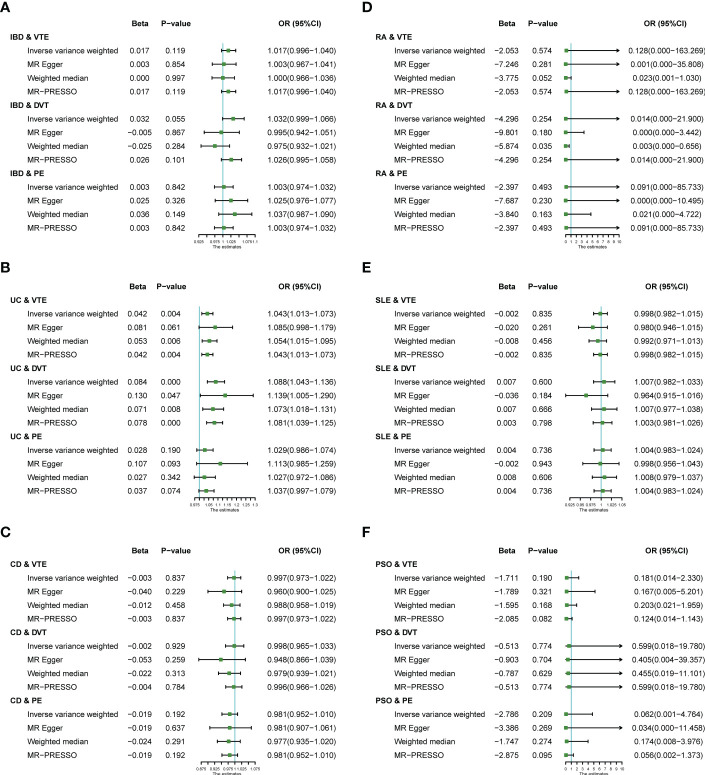
Estimates from Mendelian randomization analysis of IMIDs [**(A)** IBD; **(B)** UC; **(C)** CD; **(D)** RA; **(E)** SLE; **(F)** PSO] and risk of VTE/DVT/PE. OR, Odd Ratio; IBD, inflammatory bowel disease; CD, Crohn’s disease; UC, ulcerative colitis; RA, rheumatoid arthritis; SLE, Systemic Lupus erythematosus; PSO, Psoriasis; VTE, Venous thromboembolism; DVT, deep vein thrombosis; PE, pulmonary embolism; CI, confidence interval.

We performed MR analysis on six IMIDs diseases, including IBD and two of its subtypes (UC and CD), RA, SLE, and PSO; with VTE, DVT and PE. This allowed us to test a total of 18 causality pairs, of which two were statistically significant. As shown in **Figure 2**, the IVW model indicated that genetically predicted UC is associated with a higher risk of VTE (OR = 1.043, 95% CI: 1.013-1.073, *p* = 0.004) and DVT (OR = 1.088, 95% CI: 1.043-1.136, *p* < 0.001). The MR-Egger regression, Weighted median, and MR-PRESSO analyses showed that the IVW association pattern remained directionally consistent in most statistical models, demonstrating the robustness of the inferred causal relationships between UC and VTE/DVT (**Figure 2B**). In addition, the risk of DVT in genetically predicted IBD patients had an increasing trend with marginal statistical effect in the IVW analysis (OR = 1.032, 95% CI: 0.999-1.066, *p* = 0.055) (**Figure 2A**).

Otherwise, there was no evidence of a causal relationship between UC and PE (OR = 1.029, 95% CI: 0.986-1.074), IBD and VTE (OR = 1.017, 95% CI: 0.996-1.040), IBD and PE (OR = 1.003, 95% CI: 0.974-1.032), CD and VTE (OR = 0.997, 95% CI: 0.973-1.022), CD and DVT (OR = 0.998, 95% CI: 0.965-1.033), CD and PE (OR = 0.981, 95% CI: 0.952-1.010), RA and VTE (OR = 0.128, 95% CI: 0.000-163.269), RA and DVT (OR = 1.014, 95% CI: 0.000-21.900), RA and PE (OR = 0.091, 95% CI: 0.000-85.733), SLE and VTE (OR = 0.998, 95% CI: 0.982-1.015), SLE and DVT (OR = 1.007, 95% CI: 0.982-1.033), SLE and PE (OR = 1.004, 95% CI: 0.983-1.024), PSO and VTE (OR = 1.181, 95% CI: 0.014-2.330), PSO and DVT (OR = 0.599, 95% CI: 0.018-19.780), PSO and PE (OR = 0.062, 95% CI: 0.001-4.764) in the IVW analysis results (**Figures 2A-F**).

### 3.3 MR sensitivity analysis

In the Cochran’s Q test, p-values of Q statistics in the IBD-DVT, UC-DVT, UC-PE, CD-VTE, CD-DVT, RA-VTE, SLE-DVT, PSO-VTE, PSO-DVT, PSO-PE analyses were lower than 0.05, indicating IVs heterogeneity (**Table 2**, **Supplementary Figure S1**) and justifying the use of a random effects model in these cases. The remaining IVW analyses were performed using a fixed-effects model instead. We note that no MR-Egger regression intercepts deviated from zero (**Supplementary Figure S2**), and no evidence for horizontal pleiotropy in the IMIDs’ IVs with VTEs (all intercept *p* > 0.05) (**Table 2**). Finally, the leave-one-out analysis confirmed no causal associations were driven by a specific IV (**Supplementary Figure S3**).

**Table 2:** Heterogeneity and pleiotropy tests for the associations between IMIDs with VTE/DVT/PE.

MR analysis	nIVs	Heterogeneity test		Pleiotropy test		
		Q -*p*val	Q	Egger_intercept	SE	*p*
IBD-VTE	95	0.153	108.012	0.002	0.003	0.370
IBD-DVT	95	0.034	120.602	0.006	0.004	0.111
IBD-PE	95	0.572	90.887	-0.004	0.004	0.285
UC-VTE	51	0.065	65.950	-0.007	0.007	0.329
UC-DVT	51	0.017	73.551	-0.008	0.010	0.451
UC-PE	51	0.028	70.763	-0.013	0.010	0.186
CD-VTE	76	0.001	117.034	0.007	0.005	0.224
CD-DVT	76	0.003	113.060	0.009	0.008	0.237
CD-PE	76	0.315	80.373	0.000	0.007	0.990
RA-VTE	5	0.003	16.283	0.026	0.022	0.314
RA-DVT	5	0.071	8.644	0.028	0.022	0.299
RA-PE	5	0.126	7.194	0.027	0.020	0.277
SLE-VTE	40	0.081	51.929	0.007	0.006	0.245
SLE-DVT	40	0.012	61.734	0.017	0.009	0.077
SLE-PE	40	0.887	28.695	0.002	0.008	0.796
PSO-VTE	20	0.005	38.821	0.000	0.006	0.946
PSO-DVT	19	0.049	28.942	0.002	0.008	0.791
PSO-PE	20	0.000	54.185	0.003	0.011	0.758

nIVs, Number of instrumental variables; Q, heterogeneity statistic Q; SE, standard error; IBD, inflammatory bowel disease; CD, Crohn’s disease; UC, ulcerative colitis; RA, rheumatoid arthritis; SLE, Systemic Lupus erythematosus; PSO, Psoriasis; VTE, Venous thromboembolism; DVT, deep vein thrombosis; PE, pulmonary embolism.

## 4 Discussion

The association between inflammatory diseases and the risk of venous thromboembolism has received increasing attention from the scientific community (6, 12, 33), but to our knowledge, this is the first study to systematically explore potential causal relationships between IMIDs and VTE risk using MR methods. Our findings suggest that genetic predisposition to UC (one subtype of IBD) is associated with an increased risk of VTE and DVT. However, no MR evidence supports potential causality between genetic predisposition to CD, RA, SLE, and PSO, and the risk of VTE/DVT/PE.

IMIDs constitute a diverse and pervasive spectrum of diseases driven by immune and genetic pathways and characterized by alterations in the cellular homeostasis of the body (9). In addition to affecting various parts of the body, systemic involvement is common in different IMIDs due to abnormal activation of the immune system and inflammatory pathways, which reportedly increases the risk of venous thrombosis. However, a lack of evidence from high-quality RCT studies based on the association between IMIDs and VTE risk and poor consistency across observational epidemiological studies make these relationships unclear.

In this study, we showed a causal relationship between genetically predicted UC and VTE/DVT, as well as an elevated DVT risk in total IBD. Previous observational studies reported an increased risk of thrombosis in IBD patients (34), in accordance with our findings based on MR analysis. Furthermore, two recent cohort studies based on Asian populations reported that IBD patients are 1.80-, 1.98- and 2-fold more likely to develop PE, DVT (35), and VTE (36), respectively. A meta-analysis summarizing data from 11 observational studies covering 3,175,012 IBD patients and 920,144,253 controls showed an RR of 2.03 (95% CI: 1.72-2.39) for the occurrence of VTE in the former group (37). However, these observational studies reported significant heterogeneity, as demonstrated by stratified analysis based on sample size showing lower VTE risk in larger samples (RR 1.77, 95% CI 1.48-2.13 in large sample size studies; versus RR 2.67, 95% CI 1.97-2.93 in smaller sample sizes). These observations highlight how observational findings are susceptible to sample size and confounding factors that can be overcome using MR analysis.

The IBD GWAS dataset used in this study combines UC and CD, whereby we performed separate MR analyses on total IBD, UC, and CD. Unlike previous studies which reported that the risk of VTE was increased in both CD and UC patients (35–37), the MR evidence provided by this study only showed a causal relationship between UC and VTE. Notably, the meta-analysis results published by Fumery, M et al. showed a significantly higher risk of VTE in UC inpatients than in CD inpatients (P = 0.0029) (14), which together with our findings suggested that UC patients may have a greater risk of VTE compared with CD patients.

Our separate PE and DVT analyses showed that genetically predicted UC was causally associated with DVT only, despite evidence for increased risk for both DVT and PE in IBD patients (DVT risk is nearly twice that of PE) (38, 39). Since PE is mostly a secondary event following DVT, it is expected that there is no direct causal association between IBD and PE, which may explain this seemingly contradictory result (40).

While previous controversial associations were found for other common IMIDs (i.e., RA, SLE, PSO) and VTE/PE/DVT risk, such relationships were not apparent in our study. Galloway et al. reported an increased risk of VTE in RA patients compared to matched controls (AHR 1.54, 95%CI 1.40-1.70), but found no evidence for an association between psoriasis and VTE risk (AHR 1.21, 95%CI 0.96 to 1.52) (12). Similarly, another large RA cohort study from North America reported associations with increased risk of VTE, PE, and DVT, but this and multiple studies noted that two different JAK inhibitors used to treat RA (i.e., baricitinib and tofacitinib) were also associated with an increased risk of thromboembolism, making this an important confounder (15, 41). In addition, a long-term cohort study found a higher risk of VTE in SLE patients compared with matched control subjects. Specifically, during a median follow-up of 8.5 years, the incidence of VTE was 6.03% (95%CI: 5.17%-6.98%) in SLE patients and 1.68% (95%CI: 1.44%-1.95%) in controls (42). However, results from an MR study of SLE and cardiovascular disease conducted by Gao et al. demonstrated no causal associations exist between SLE and VTE risk (OR=1.001, 95% CI [1.000-1.002]), which is consistent with our findings (20).

Despite extensive evidence from observational studies on the relationship between IMIDs and VTE risk, these approaches have obvious limitations and confounding factors, such as an inability to evaluate causal relationships between exposure and outcome. In addition, with glucocorticoids, immunosuppressants, and antithrombotic drugs widely used in the treatment of IMIDs, the role of these drugs in promoting or inhibiting thrombosis has become an important confounder in determining the relationship between IMIDs and the risk of VTE (9). For example, high doses of glucocorticoids significantly increase the risk of atherosclerosis and thrombosis (43, 44); immunosuppressants including azathioprine, cyclophosphamide, and anti-tumor necrosis factor drugs may help suppress thrombosis by controlling systemic inflammation and disease activity (43); interestingly, some immunosuppressive agents such as JAK inhibitors have been reported to have some adverse risk of thrombosis in RA treatment (45). The use of these drugs to inhibit or promote VTE in IMIDs has inevitably resulted in confounding bias in observational studies. By calculating the correlation between the genetically predicted IMIDs risk and the genetically predicted VTE risk, MR Study cleverly reduced the influence of confounding deviation in the intermediate process. This stresses the importance of using Mendelian randomization strategies to explore the risk of VTE in patients with IMIDs.

Our study is the first MR analysis to explore potential causal relationships between multiple IMIDs diseases (IBD, UC, CD, RA, SLE, PSO) and VTE risk (including DVT and PE). When compared to previous observational studies, our MR design circumvents traditional confounding factors and problems associated with reverse causation. Moreover, we tested these relationships on populations with the same ethnicity using large-scale GWAS data, which provides strong and reliable IVs and strengthens inferences of causality that were confirmed by sensitivity analyses. However, a lack of demographic data (e.g., sex and age) in the original study hampered further subgroup analyses. In addition, MR analyses were not performed in disease subcategories – for example, psoriasis included psoriasis-associated arthritis, which was not evaluated separately for an association with VTE risk. Due to the small number of available GWAS for our study subjects, we were unable to perform multi-database validation. Finally, it should be noted that GWAS data was used for people of European ancestry, whereby extrapolations to other ethnic groups are limited.

## Conclusion

This MR study provides new genetic evidence for the relationship between IMIDs and VTE risk. We found the existence of a causal association between UC and VTE/DVT risk, but no evidence for causal associations with other IMIDs, including CD, RA, SLE, and PSO. Our findings highlight the importance of active intervention and monitoring to mitigate VTE risk in patients with IBD, especially those presenting with ulcerative colitis. Future longitudinal clinical studies and experimental analyses are needed to confirm our findings.

## Data availability statement

The original contributions presented in the study are included in the article/**Supplementary Material**. Further inquiries can be directed to the corresponding author.

## Author contributions

JW designed, guided, and funded the study. XL conducted most of the MR analysis and draft of the manuscript. XG draft the manuscript and prepare the figure and Table. JL, QN and YD conducted data acquisition and provided technical support. XF, ZY and PL critically revised the manuscript. All authors contributed to the article and approved the submitted version.

## Supplementary material

Supplementary Table 1 The detailed characteristics of IVs.
