# Supplementary material for: Immune-mediated inflammatory diseases and risk of venous thromboembolism: A Mendelian randomization study
Source: Front Immunol. 2022 Dec 13;13:1042751. doi: 10.3389/fimmu.2022.1042751 (PMC9792973; doi:10.3389/fimmu.2022.1042751)
Supplement: Supplementary Table 1 — The detailed characteristics of IVs. [file DataSheet_1.docx]

Supplementary Material


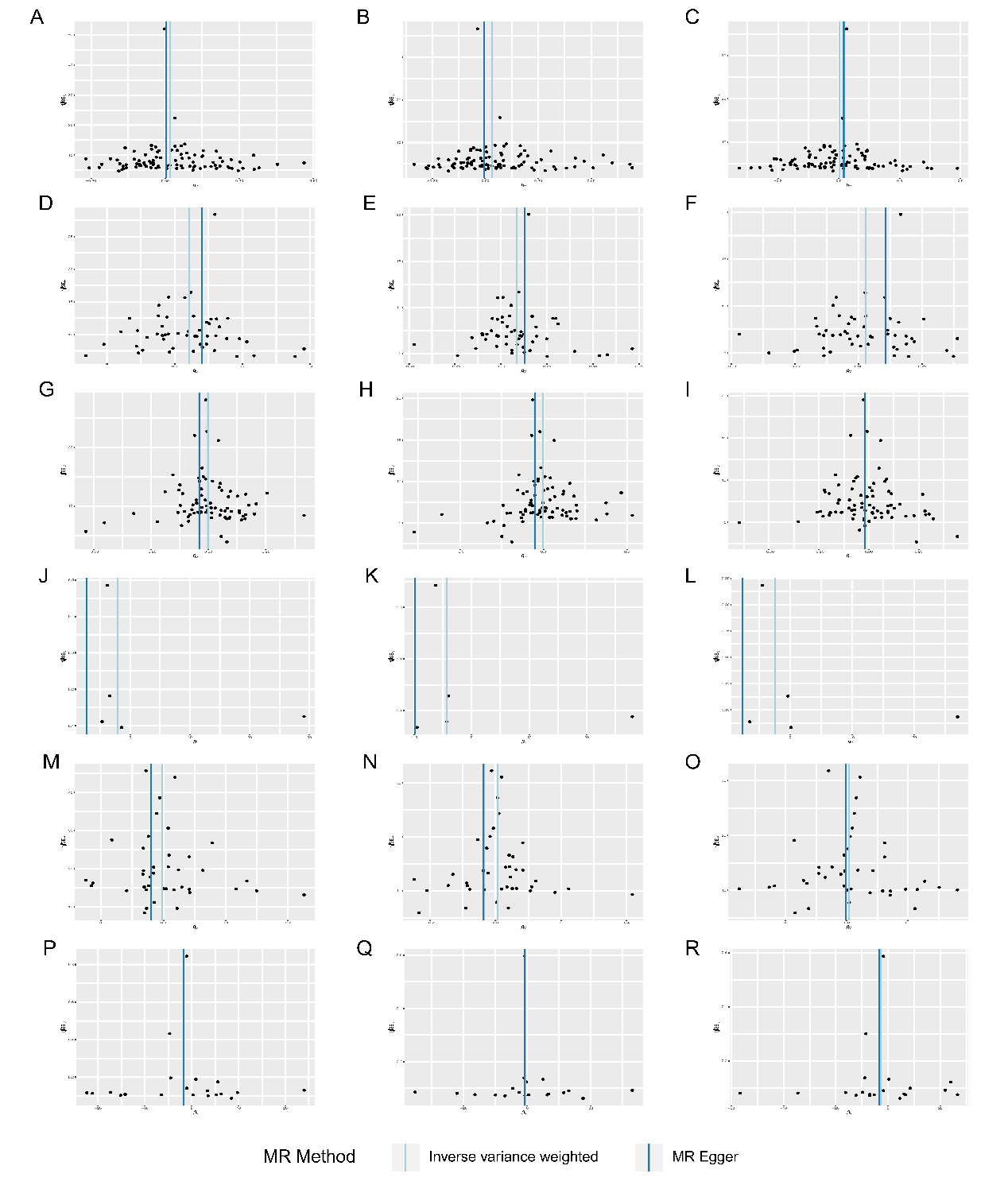


**Supplementary Figure 1.** Funnel plot for IVW and MR-Egger method. (A) IBD and VTE; (B) IBD and DVT; (C) IBD and PE; (D) UC and VTE; (E) UC and DVT; (F) UC and PE; (G) CD and VTE; (H) CD and DVT; (I) CD and PE; (G) RA and VTE; (K) RA and DVT; (L) RA and PE; (M) SLE and VTE; (N) SLE and DVT; (O) SLE and PE; (P) PSO and VTE; (Q) PSO and DVT; (R) PSO and PE.


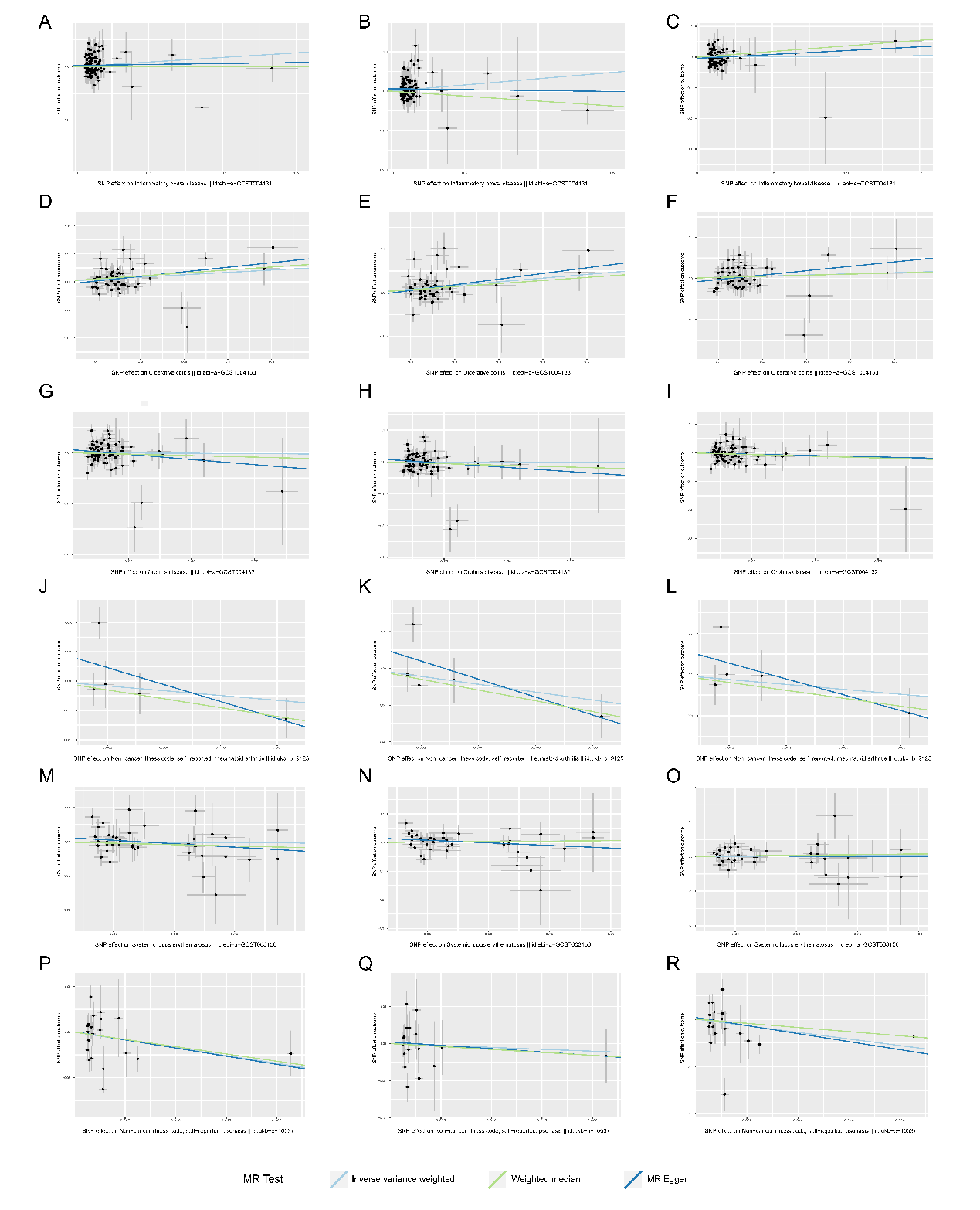


**Supplementary Figure 2.** Scatter plot using all IVs. (A) IBD and VTE; (B) IBD and DVT; (C) IBD and PE; (D) UC and VTE; (E) UC and DVT; (F) UC and PE; (G) CD and VTE; (H) CD and DVT; (I) CD and PE; (G) RA and VTE; (K) RA and DVT; (L) RA and PE; (M) SLE and VTE; (N) SLE and DVT; (O) SLE and PE; (P) PSO and VTE; (Q) PSO and DVT; (R) PSO and PE.


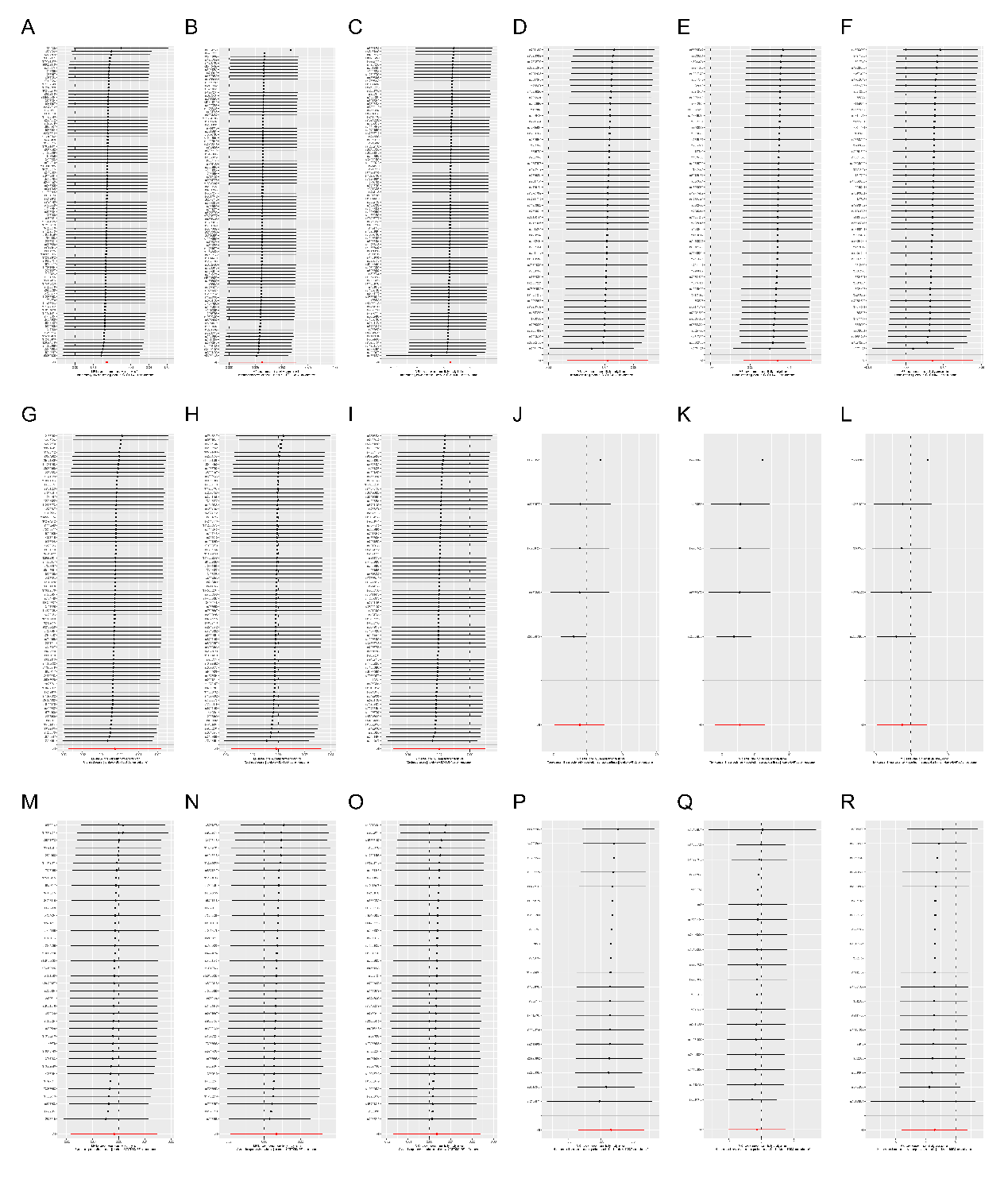


Supplementary Figure 3. Leave-one-out sensitivity analysis. (A) IBD and VTE; (B) IBD and DVT; (C) IBD and PE; (D) UC and VTE; (E) UC and DVT; (F) UC and PE; (G) CD and VTE; (H) CD and DVT; (I) CD and PE; (G) RA and VTE; (K) RA and DVT; (L) RA and PE; (M) SLE and VTE; (N) SLE and DVT; (O) SLE and PE; (P) PSO and VTE; (Q) PSO and DVT; (R) PSO and PE.
